# Normalization of a chromosomal contact map

**DOI:** 10.1186/1471-2164-13-436

**Published:** 2012-08-30

**Authors:** Axel Cournac, Hervé Marie-Nelly, Martial Marbouty, Romain Koszul, Julien Mozziconacci

**Affiliations:** 1 LPTMC, UMR 7600, Tour 12-13/13-23, Boîte 121, 4, Place Jussieu, 75252 Paris Cedex 05, France; 2 Institut Pasteur, Groupe Imagerie et Modélisation, Department of Cellular Biology and Infection, F-75015 Paris, France; 3 CNRS, URA2582, F-75015 Paris, France; 4 University Pierre et Marie Curie, Cellule Pasteur, 75252 Paris Cedex 05, France; 5 Institut Pasteur, Spatial regulation of genomes group, Department of Genomes and Genetics, F-75015 Paris, France; 6 CNRS, UMR3525, F-75015 Paris, France

## Abstract

**Background:**

Chromatin organization has been increasingly studied in relation with its important influence on DNA-related metabolic processes such as replication or regulation of gene expression. Since its original design ten years ago, capture of chromosome conformation (3C) has become an essential tool to investigate the overall conformation of chromosomes. It relies on the capture of long-range trans and cis interactions of chromosomal segments whose relative proportions in the final bank reflect their frequencies of interactions, hence their spatial proximity in a population of cells. The recent coupling of 3C with deep sequencing approaches now allows the generation of high resolution genome-wide chromosomal contact maps. Different protocols have been used to generate such maps in various organisms. This includes mammals, drosophila and yeast. The massive amount of raw data generated by the genomic 3C has to be carefully processed to alleviate the various biases and byproducts generated by the experiments. Our study aims at proposing a simple normalization procedure to minimize the influence of these unwanted but inevitable events on the final results.

**Results:**

Careful analysis of the raw data generated previously for budding yeast *S. cerevisiae* led to the identification of three main biases affecting the final datasets, including a previously unknown bias resulting from the circularization of DNA molecules. We then developed a simple normalization procedure to process the data and allow the generation of a normalized, highly contrasted, chromosomal contact map for *S. cerevisiae*. The same method was then extended to the first human genome contact map. Using the normalized data, we revisited the preferential interactions originally described between subsets of discrete chromosomal features. Notably, the detection of preferential interactions between tRNA in yeast and CTCF, PolII binding sites in human can vary with the normalization procedure used.

**Conclusions:**

We quantitatively reanalyzed the genomic 3C data obtained for *S. cerevisiae*, identified some of the biases inherent to the technique and proposed a simple normalization procedure to analyse them. Such an approach can be easily generalized for genomic 3C experiments in other organisms. More experiments and analysis will be necessary to reach optimal resolution and accuracies of the maps generated through these approaches. Working with cell population presenting highest levels of homogeneity will prove useful in this regards.

## Background

Chromosomes from both eukaryotes and prokaryotes not only convey information through their linear DNA sequence but also contribute to the regulation of a number of DNA-related metabolic processes through their three dimensional arrangements [1-3]. Since an original publication by Dekker and co-workers ten years ago, chromosome conformation capture (3C) technique and its derivatives have become essential to the investigation of chromosome organization [4-6]; for a brief overview of the various techniques published so far see [7]. The general principles of these protocols remain the same and rely on formaldehyde fixation to capture long-range trans and cis chromosomal interactions in living cells. The crosslinked cells are incubated with a restriction enzyme that will cut the DNA in a number of restriction fragments (RFs). Because of the crosslink, several RFs can be covalently linked within molecular complexes. A ligation step in diluted conditions will favor ligation events between RFs trapped within the same complex. After a decrosslinking step, the resulting 3C template consists in a collection of ligation products of two specific RFs, whose relative abundance (after normalization) reflects the frequency with which these two chromatin segments were crosslinked in the population. The exhaustive analysis of this collection enables the generation of chromosomal contact maps, that allows deciphering the average positioning of loci of interest with respects with each others within the nucleus. In the past few years, quantification of the abundance of ligation products has evolved from semi-quantitative PCR [4] to deep-sequencing techniques [8]. The later approach now enables genome-wide analysis of chromosome organization. A typical result of such experiment is the number of times each pair of RF is sequenced at the final step. These numbers are then arranged in a symmetric matrix representing all the possible pairs of RFs from the genome, generating a genome-wide contact map. Those matrices represent the relative frequency of physical interaction for each RF in the genome with all of the other RFs. Different experimental protocols have been used so far, and genome-wide contact maps have been obtained for Lymphoblastoid cells [8,9], mouse [10,11], *Schizosaccharomyces pombe*[12], *S. cerevisiae*[13,14], and fruit fly [15].

**Figure 1:**
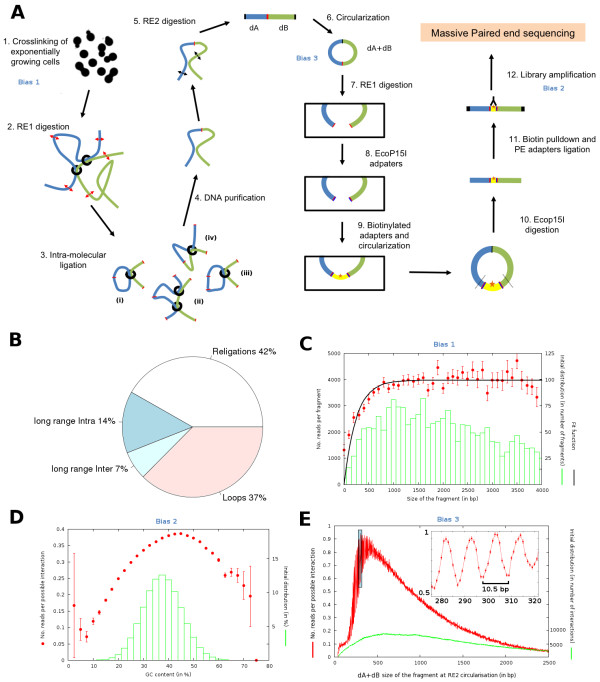
**The different steps of the original genomic 3C experiment in yeast and their associated biases **[13]**. A**) Experimental steps. 1: Yeast cells are fixed with formaldehyde. 2: the genome is digested using a 6 cutter restriction enzyme (RE1; red double-headed arrows). 3: extraction of protein/DNA complexes and ligation in diluted conditions that favor DNA-end interactions and religation within the same complex. During this process, some RF will simply circularize (i), while others will religate in their original orientation (ii). Religation products are also expected between non-collinear restriction fragments (iii), whereas collinear RF separated by one, or more, RF will also interact together (iv). 4: de-crosslinking and DNA purification. 5: digestion of DNA products using a frequent 4 cutter restriction enzyme (RE2; black double-headed arrows). 6: DNA is ligated in diluted conditions, favoring intra-molecular circularization of single DNA molecules. Remaining linear fragments are degraded. 7: DNA circles containing a RE1 site are re-opened using RE1. 8: short DNA sequences, containing EcoP15I recognition site and a biotinylated nucleotide are added at both ends of the linear fragments. 9: circularization of linear fragments. 10: EcoP15I digestion of the DNA segments 25 bp apart from the enzyme recognition site. 11: pull-down of the DNA fragments containing biotinylated nucleotides. 12: amplification of the DNA fragment isolated and sequencing. **B**) Pie-chart representation of the different types of events obtained at step 3: religations, long range intra, long range inter, loops (from 50 millions pair-end sequences analyzed from the HindIII-MspI condition A and B experiments). **C**) Quantification of the fragment length bias. **D**) Quantification of the GC bias. **E**) Quantification of the circularization length bias.

3C derived experiments are likely to generate biases given the complexity of the protocols, and necessitate a dedicated effort to experimentally identify and limit the generation of byproducts at each step [16]. However, it appears impossible to entirely prevent unwanted DNA molecules to be present in the final banks, and subsequently in the sequence data. Therefore, these data need to be carefully processed in order to identify these sequences, and limit the introduction of biases in the final analysis. Although not necessarily rewarding, such (re-)processing is essential not only to accurately analyze the data from a specific experiment but also to provide important feedback for the design of future experiments. For instance, GC content and RF lengths induced biases present in the Hi-C databank of the Human genome were recently identified [17]; see also [18]. Here, we have reassessed the genomic 3C data from the experimental protocol used to obtain the first comprehensive dataset in *S. cerevisiae* in a pioneering study published recently (Figure 1A; [13]). Using HindIII as 3C restriction enzyme, the interactions between 4454 sites along the 12 Mbp yeast genome were mapped and a symmetric matrix of 4454 rows per 4454 columns was generated. A number of interesting features, some of them expected, such as centromere clustering resulting from the Rabl configuration, and others less obvious, such as early replication origins clustering, were identified from this matrix [13]. Interestingly, the re-analysis of the raw data obtained through this protocol lead to the characterization of a number of events and biases unidentified before. Back-and-forth comparison between these biases and the protocol steps allowed us to identify the different sources for these events.

Having properly identified and quantified all these biases, we developed a normalization procedure which allows us to correct the data for all those biases at one time. Overall, and as expected from the original analysis, the conclusions drawn from the corrected maps do not differ significantly from the original publication. However, the corrected map gives a more contrasted view of chromosomal contacts, and present sharper features when it comes to preferential interactions between telomeres or chromosomal arms. It also ponders some of the conclusions drawn regarding clustering of specific genetics elements, which will be discussed. We then used this approach on the genomic 3C (Hi-C) human dataset obtained by Dekker and co-workers [8] and showed that proper normalisation is a prerequisite to assess relevant contacts. The methodology described here allows for an efficient and simple analysis of chromosomal contact-maps, and is potentially of great convenience to any team interested to use similar approach.

## Results and discussion

### Quantification of the ligation products

During the ligation step, one can envision to recover different types of products (Figure 1A, step 3). Firstly, a RF can simply be circularized on itself (step 3i), resulting in a loop. Secondly, two consecutive RF on the genome can be re-assembled together (step 3ii). This type of event will be designated as a religation event. Note that religation events are virtually indistinguishable from non-digested restriction site (RS) given the original sequence is then restored. A third type of product can be recovered at this step, especially if the digestion is partially incomplete which will always be the case: longer DNA fragments formed out of two continuous RFs can be circularized during the ligation step (step 3iii). Finally, two RFs that are not consecutive on the genome can be ligated together (step 3iv). These products are the nuggets the experiment is digging for, and will be termed here as long-range interactions. Long-range interactions can either be intra or inter-chromosomal. Although inter-chromosomal events are easily identified through mapping of the pair-end reads along the genome, intra-chromosomal events necessitate a more careful examination of the positions of the sequences. A convenient way to identify the type of an intra-chromosomal ligation product is to use the orientation of the sequences obtained from the pair-end sequencing run. Each RF exhibits two extremities. The one with the highest coordinate according to the yeast genome conventional representation is labeled “+” and the other one “-”. Every ligation event therefore falls within one of these four categories: -/-, +/+, -/+ and +/- (see Additional file 1: Figure S1A). Whereas long range interactions should not happen with any preferential orientation of the fragment extremities, a circularized RF will always connect its – extremity with its + extremity (Additional file 1: Figure S1A). The distribution of interaction types (+/+, -/-, -/+ and +/-) can be plotted for self-interacting fragments as well as for contiguous fragments (i.e. separated by only one RS), and then separated by two, and more RSs. For the later category no preferential orientations are distinguishable (Additional file 1: Figure S1B). A strong enrichment in +/- interactions is observed for pairs of collinear RFs. This enrichment is due to the presence of religation events (ii) as well as detection of sites which escaped the digestion step. The formation of type (iii) products is revealed by the fact that interactions between contiguous fragments on the genome are more often found in the -/+ configuration, which corresponds to a loop, than in a -/- or +/+ configuration. The relative number of those different products can be represented with a pie chart (Figure 1B). Loops and religation appear to be very frequent events (about 80% of the original data). Those inevitable byproducts were removed from all subsequent analysis. In addition, fragments with no restriction site for the secondary enzyme and therefore that should not be detected according to the experimental protocol were also discarded. Similarly, fragments whose extremities align ambiguously along the reference genome were removed as well (see Methods for details). In total, more than 80% of the initial raw reads were removed for subsequent analysis, which is consistent with other experiments in the field, and leaves room for a lot of improvement.

### Identification of major biases in the experimental protocol

Complex protocols involving a large number of steps are likely to generate biases in the data that has to be careful sought for. What we call biases here is a variability which is larger than the expected noise and can be explained primarily by properties of the fragment itself. In the following, three major biases likely to affect the number of detected interactions between fragment pairs were identified: the length of RFs, GC content of the paired-end reads, and the length of DNA segments at the circularization of steps 6 and 9.

The distribution of the number of reads per fragment as a function of the fragment size *L* is presented on Figure 1C. Given the number of positions accessible to fixating agents along a RF increases with its size, one would expect the interaction probability to increase linearly with RF size. For RF under 800 bp, the number of reads per fragment increases, suggesting that indeed the probability for a cross-linking event to occur depends on the length of the fragment. However, for longer RFs, a plateau is reached, suggesting that the maximum probability for at least one cross-linking event to occur along that length is reached. In other words, the probability of longer fragments not to be cross-linked at least once is constant and very small (Methods).

Formaldehyde fixation, which is the first step of 3C based protocols, therefore introduces a length bias for sizes under 800bp. In this range, the longer a RF is, the more likely it will be cross-linked with other RF during the fixation step.

The distribution of reads per possible interaction between two RF extremities was plotted as a function of the GC content of these extremities (Figure 1D). From this figure one can see that extreme GC content extremities tend to be under represented in the final interaction reads. Therefore, the PCR reaction or/and the deep-sequencing steps can introduce additional biases, notably by favoring reads with a GC content of about 45%. The bias of GC content in short reads data from high-throughput DNA sequencing has indeed been reported (see Figure 2 in [19]). However, such biases do not appear to affect many interactions (see Figure 1D).

**Figure 2:**
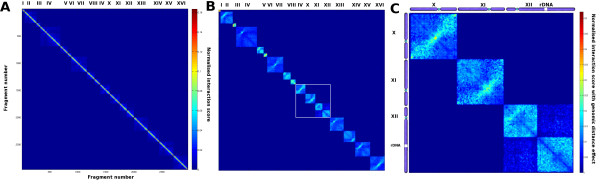
**Normalized intra-chromosomal contact map of *S. cerevisiae*.** The color scale represents the normalized interaction frequencies between fragments which is calculated with the Sequential Component Normalization. **A**) Matrices of the sixteen chromosomes from *S. cerevisiae*. The strongest interactions are at the diagonale i.e. for close fragments along the chromosome. **B**) The normalized interaction score is calculated with the SCN method and taking into account the effect of the genomic distance. **C**) Zoom on chromosomes X, XI and XII. Chromosome XII is spatially segregated in two compartments by the rDNA locus.

Quite surprisingly we also identified an original, but retrospectively not unexpected, bias in the two steps involving circularization of DNA segments (Figure 1A, step 6 and 9). It is known that the mechanical properties of DNA are such that the length of a fragment can strongly influence the efficiency of a circularization reaction. If the fragment is too small, the bending persistence of DNA is such that both ends cannot be ligated. If the fragment is two long, the entropic contribution to the free energy will also disfavor ligation. Here indeed, the distribution of the sum of the sizes (*d*_*A *_+* d*_*B*_ ) of two interacting RF A and B presents a typical circularization efficiency profile, including an optimal circularization length close to 500 bp (Figure 1E, [20]).

Intriguingly, a 10.5 bp periodicity of the circularization efficiency could be observed for the average number of circularization events for which *d*_*A *_+ *d*_*B *_< 500 bp, overall (i.e for the HindIII-MspI experiment, about 15% of the interactions fall into this category). Such a periodicity is actually predicted by polymer physics and results from the natural twist of the double helix which is 10.5 bp [21]. Here, the phenomenon can be observed at an unprecedented resolution (see inset of Figure 1E) and consists in a bias that could affect any experimental procedure involving a circularization through ligation step.

Due to those various biases, some RFs will be involved in more interactions than expected, whereas others will be underrepresented in the final bank (see Additional file 1: Figure S2). Since this variability results from the experimental protocol rather than the biological reality, it is worth minimizing theses effects by either correcting or normalizing the observed frequencies of interactions [17,18]. These correspond to two different approaches: in order to correct the data, one needs to quantify the biases and then to divide each interaction frequency by its expected value, knowing the bias. On the other hand, no prior knowledge of the bias is needed to normalize the data: the procedure consists in dividing each interaction frequency between two fragments by the product of the sums, or the norms, of the total interaction reads involving those fragments (see below).

### Generation of a normalized contact map through the “Sequential Component Normalization” (SCN) methodology

The correction method developed for the human Hi-C dataset is not readily adaptable to the yeast dataset since there is an additional circularization bias to the RF length and GC content bias [17]. A important issue with the circularization bias is that it is highly non monotonous: for example, it favors circularization lengths of 261 bp, but disfavors circularization length of 266 bp and again favors circularization lengths of 271 bp and so on and so forth (see inset in Figure 1D). A similar methodology that was previously described in [17] was first applied in order to correct for this bias. However, the nature of the bias did not allow reaching a satisfying solution because of the non-monotonous specificity. In the following, instead of correcting each of the interactions frequencies individually, contact maps were normalized globally through what we called the SCN approach, which can be applied to any genomic contact map and independently from the protocol that was used to generate it. The normalization described below is based on the interactions exhibited by the entire restriction fragments, before the second digestion, in order to remain as broadly generalizable as possible to other experimental protocols. The reason why we applied normalization on the fragment instead on the extremities is that for each pair of fragment there are four possibilities to make religation event. Each of those four possibilities will exhibit a different GC content and a different dA+dB and therefore the biases described in Figure 1D and 1E, that depends on the extremities, will be smoothen out when aggregating the combinations together. This point was also discussed in the original paper [13]. The advantage of this method is that it smoothens out all the biases described above and therefore provides a cleaner view of the frequency of interaction between any pair of restriction segments in the genome.

Intra- and inter-chromosomal interactions were treated separately but using the same procedure. Firstly, normalization will give an equal weight to each fragment in the contact map. Therefore, RF with very low number of reads, corresponding to RF that could not be properly detected, are likely to introduce noise in the normalized contact map and have to be removed (see Additional file 1: Figure S3). In order to identify these fragments, we computed the distribution of reads in the contact map (see Additional file 1: Figure S2B). This distribution is roughly gaussian, with a long tail corresponding to low interaction fragments. Based on this distribution, we cut the tail of the distribution (see Methods for further information).

**Figure 3:**
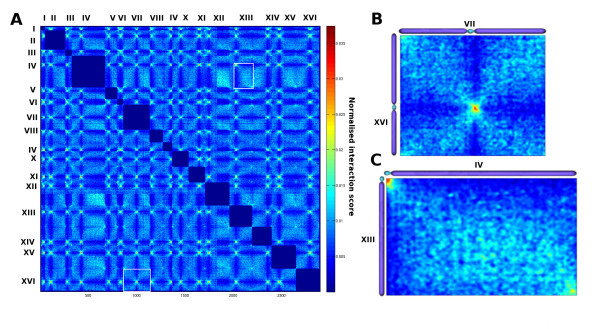
**Normalized inter-chromosomal contact map of *S. cerevisiae*.** The color scale represents the normalized interaction frequencies between fragments which is calculated with the Sequential Component Normalization. **A**) Matrix of the sixteen chromosomes from *S. cerevisiae*. **B**) Zoom on chromosomes VII and XVI. **C**) Zoom on chromosomes IV and XIII.

Once low interacting fragments are removed, we wish to normalize all rows and columns of the contact map to one so that the matrix remains symmetric. This was done through the following simple procedure. Firstly, each column vector was normalized to one, using the euclidian norm. Then each line vector of the resulting matrix was normalized to one. The whole process was repeated sequentially until the matrix become symmetric again with each row and each column normalized to one (Additional file 1: Figure S4 and Methods). Usually, two or three iterations are sufficient to insure convergence. Since it involves a sequential normalization of column and line vectors of the matrix, this method was named Sequential Component Normalization (SCN). This normalization can be viewed as a sequence of extensions and shrinking of interaction vectors so that they tend to reach the sphere of radius one in the interactions space. A similar and faster approach is to divide all the matrix elements *c*_*ij*_ by the product of the norms of row *i* and column *j* : $c_{\text{ij}}^{*}=\frac{c_{\text{ij}}}{\left|c_{\text{ik}}||c_{\text{kj}}\right|}$. This method yields to a normalized contact map overall very similar to SCN (Additional file 1: Figures S5 and S6). However since the sum of each component is not necessarily equal using this method, it may bias further analysis such as assessing the 3D colocalization of genomic elements (see below). An alternative normalization method has been used so far by other groups [9], that use the sum of the components instead of the euclidian norm : $c_{\text{ij}}^{*}=\frac{c_{\text{ij}}}{\underset{k}{\sum }c_{\text{ik}}\underset{k}{\sum }c_{\text{kj}}}$. We noticed that this method yields to a contact map with lower contrast than the SCN (Additional file 1: Figure S5 and S6) and therefore recommend SCN use in further works. The normalization using the sum will give more weight to fragments wich makes fewer interactions whereas our normalization will give more weight to fragments interacting moderately with many fragments. Intra and inter-chromosomal interactions were separated in two datasets and the corresponding normalized contact matrices between RFs were plotted as a function of their position along chromosomes (Figure 2A and 3A, respectively).

### *S. cerevisiae* contact maps after SCN

The normalized maps overall are similar to those observed before [13]. Since the probability of interaction between monomers along a polymer is decreasing with the linear distance between them, the diagonal which represents neighboring RFs presents the highest interactions score [4]. In order to increase the contrast and observe interactions between non-adjacent intra-chromosomal RF we then divided the number of interactions between fragments separated by a genomic distance *D*_*g *_by the average interaction count between fragments separated by the same distance *D*_*g*_ (see Methods). Some features appear more contrasted with respect to the original analysis, with a typical *X* shape pattern centered on the centromere for each chromosome (Figure 2B). This pattern reflects the fact that the centromere does not interact much with the chromosome arms whereas both arms can interact together. In addition, interactions between RF located on both arms appear clearly more constrained when at symmetrical distances from the centromere and within its vicinity (Figure 2C). In addition, the bipartite structure of chromosome 12 due to the insulating presence of the nucleolar rDNA repeats remains clearly apparent [13]. The corrected contact maps for inter-chromosomal interactions also reveal striking features (Figure 3A). Centromere clustering is clearly apparent and results in all the centromeres interacting with each other’s on the map, as in [13]. The interactions between two chromosome arms along their length are also extremely clear. The *X* shaped patterns at inter-centromeric interactions observed in the matrix indicate that centromeres are somehow isolated from the rest of the chromosomal arm sequence (see for instance chromosome VII and chromosome XVI on Figure 3B). This feature is even more striking when the correlation matrix is drawn similarly to [8] (Additional file 1: Figure S7). In this matrix, each element *c*_*ij*_ is the Pearson coefficient between the vectors i and j.

**Figure 4:**
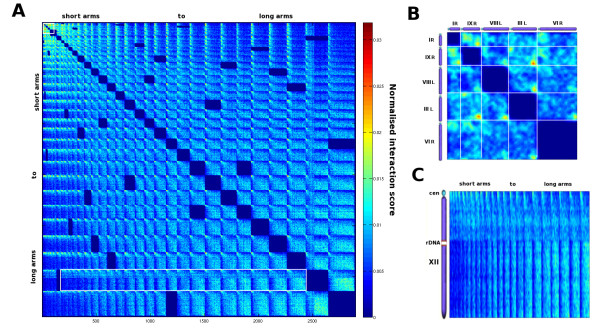
**Normalized inter-chromosomal contact map of *S. cerevisiae*.A**) Inter-chromosomal contact map of chromosomal arms ranked according to their size, from the shortest (left) to the longest (right). The white empty squares correspond to specific emphasis on the five shortest arms (**B**), and on chromosome XII (**C**).

In addition, telomeres are also found to have enriched contact frequencies (for instance chromosome XIII and chromosome IV on Figure 3C). To investigate the role of the chromosomal arm length in the inter-chromosomal interaction frequencies, all chromosomal arms were ranked with respect to their length and the corresponding contact maps were drawn (Figure 4A). This layout conveniently reveals global interaction patterns in respect to chromosomal arm size: shorter arms tend to interact with shorter arms whereas longer arms tend to interact with longer arms (from the upper left corner to the lower right corner). On the contrary, shorter arms tend to make very few contacts with longer ones (upper right and lower left corners on Figure 4A). Zooming on the five shorter arms on the contact map reveals that the interaction frequencies between subtelomeres from shorter arms are important, sometimes even more than centromeres (e.g arms III-L and IX-R, see Figure 4B). To investigate the arm length relationship with sub-telomere interactions, we computed the mean interaction frequencies between all sub-telomere pairs for both the normalized and original data. The normalized data exhibit two types of preferred subtelomeric interactions, one for short and one for long chromosome arms, whereas the orginal analysis mostly emphasized short arms interactions (see Additional file 1: Figure S8). Given that the measurements reflect a population average, it is impossible to know from this data if all the telomeres interact preferentially in a similar ways in all cells taken individually. However, similar preferred interactions have been observed in single cells using fluorescent microscopy approaches [22,23] as well as in recent modeling approaches [24]. In addition, the rDNA now appears not only as an intra-chromosomal insulator region, but also modifies the interacting properties of the two DNA segments it delimits. Whereas a gradual shift in interaction frequencies from centromere to telomere is observed for long arms, for chromosome 12 the DNA segment located between the rDNA and the telomere seems less constrained that the one before the rDNA cluster (Figure 4C).

### Re-assessing the 3D colocalization of genomic elements

The influence of this normalization procedure on the preferential interactions detected previously was addressed. In the original analysis, receiver operating curve (ROC) confirmed an expected enrichment of interactions for centromeres and telomeres resulting from the Rabl configuration [13,23]. More interestingly, early replication origins [25] were also shown to interact preferentially, a result experimentally supported [3]. Finally, two preferential interactions regions where identified for tRNA genes, one around the spindle pole body (SPB) and one in the vicinity of thenucleolus [13].

**Figure 5:**
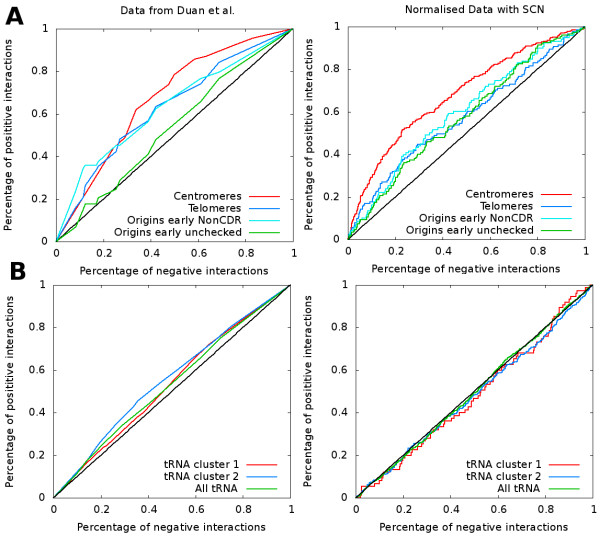
**Receiver operating curves to assess 3D colocalization of genomic elements for the yeast contact map.** Receiver operating curves (ROC) were used to assess 3D colocalization of different genomic elements. Data from Duan et al. [13] (left column) and normalized data (right column) were used. **A**) Centromeres, Telomeres, early origins of replication give positive signal with both types of data. **B**) The group of tRNA was assessed for 3D colocalization. Two clusters proposed by [13] were assessed with both data: cluster 1 of tRNA genes proposed to colocalize near rDNA and cluster 2 of tRNA genes proposed to colocalize near centromeres. The data from [13] give a positive signal contrary to the data normalized with SCN.

In this paper, we used a different method than the originally published ROC analysis. The initial ROC analysis asked the question: among the pool of strong interactions, is there an enrichment in interactions between two fragments which both carry the genomic object of interest. We ask the question: among the pool of strong interactions carrying one feature of interest, is there an enrichment for interactions with a fragment carrying the same feature (for details about the implementation, see Methods). ROC analysis on the normalized data confirmed the expected centromeres and telomeres preferential interactions (see Figure 5A). In addition, enrichment in interactions between early replication origins was also observed. However, the frequencies of interactions between restriction fragments containing tRNA genes did not exhibit significant increase when using the normalized data (Figure 5B, compare the right panel with the left panel). This was found to be true for all RFs containing tRNAs or for RFs containing only tRNAs previously found to interact preferentially with the SPB or with the nucleolus (see Figure 5B).

The previously described preferential interaction between tRNA genes was lost because it resulted from the fact that, without normalization, two fragments interacting overall more with the whole genome will interact together more frequently than other fragments. This is actually the case for tRNA fragments (see Additional file 1: Figure S9). The reason why tRNA bearing RF interact more frequently than others with all other fragments does not depend on their size, and remain open. A local improvement in cross-linking efficiency resulting from the chromatin state and/or presence of protein complexes is a possibility. Of course, we do not exclude the possibility of actual preferential interactions between tRNA as observed experimentally [26,27] and suggested by other approaches [24]. However, more experiments and higher resolution will be needed to detect those through genomic 3C approaches.

### Normalization of the human genome contact map using SCN

**Figure 6:**
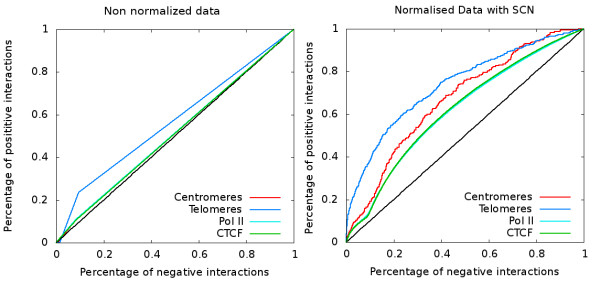
**Receiver operating curves to assess 3D colocalization of genomic elements for the human contact map.** Receiver operating curves (ROC) were used to assess 3D colocalization of different genomic elements for the human contacts map of Lieberman *et al*[8]. Non normalized data (left column) and normalized data (right column) were used. Only Telomeres give positive signal when using the non normalized data (curves for Centromeres, PolII are superimposed with the CTCF curve). When using the data normalized with SCN, all genomic elements tested give positive signal to the ROC test (curve for PolII is superimposed with CTCF curve).

In order to test how the SCN approach can be applied to the interaction map of a larger genome, we used the human genome-wide dataset published in 2009 by Lieberman et al. [8]. The restriction enzyme used in this dataset cuts the human genome over 830,000 times. Therefore, the number of potential interaction in the experiment is higher than 340 billion. Since the typical number of reads obtained in such experiment hardly reaches one billion [11], the resulting genome wide contact matrix is very sparsed. In order to get enough information to build a contact map, one can bin the matrix by adding the contacts over several fragments along the genome together. For intra-chromosomal interactions, a typical bin size of about ten fragments is adequate since most of the interaction detected in such an experiment are intra-chromosomal and since the number of possible intra-chromosomal interactions is much lower than the number of possible inter-chromosomal interactions. For inter-chromosomal interaction the bin size has to be increased considerably. We used a bin of one hundred fragments to build the corresponding contact map for the human genome and normalized it through the SCN method. The resulting map clearly shows preferential interactions between small chromosomes and between the long arm of long chromosomes (Additional file 1: Figure S10). Importantly, ROC curves which are used to determine the genomic elements enriched at high interaction hotspot strongly depend to whether or not the data were normalized. We performed ROC analysis on the binding sites of the CCCTC-binding factor (CTCF), a zinc finger protein that plays an important role in the organization of chromatin by mediating inter and intra-chromosomal contacts between distant loci [28,29], PolII, the centromeres and the telomeres. The results for both raw and normalized data clearly show that the preferential interactions of CTCF, PolII and centromeres are only seen on the properly normalized data (Figure 6).

## Conclusions

The method described above consists in an easy and convenient way to normalize and represent genomic 3C data. It is worth recalling that before doing any normalization procedure, one has to identify the products and filter out all those that do not correspond to what is expected from the experimental protocol. It represents here more than 90% of the total reads. Depending on the protocol used, the biases in the data will vary, generating an extra number of reads that should not be used in the analysis. Among those identified in the present study, the original circularization bias is certainly of importance for any experimental protocol involving a similar step. While increasing contrast and visibility of the Rabl yeast genome organization, the procedure described here confirms the preferential interactions of specific elements, such as early replication origins. However, it also revealed that what could appear like enrichment in interactions between other elements has to be carefully interpreted.

The SCN normalization procedure proposed here will be helpful once higher density contact maps of *S. cerevisiae* become available, and can be conveniently adapted to any other organisms. Increasing the resolution of these contact-maps will likely reveal more features, and can be addressed either through alternative protocols addressing the “invisible” zones of the genome (for instance by increasing the length of the sequenced reads or using various restriction enzymes), or through increasing the number of reads.

## Methods

### Alignment of the reads on the reference genome

The paired-end sequence reads from banks (SRP002120) were aligned along the yeast genome of the sequenced strain S288C (2011-02) with Bowtie2 [30]. Raw data were converted into fastq files and sent to the aligner. Only reads exhibiting non-ambiguous alignment on the genome were retained. This was done by using the preset parameter ”–very-sensitive” and setting a threshold on the mapping quality. The mapping quality Q is defined as Q = -10 × log10(p) where p is the probability that the reported position is false. The higher Q, the more unique is the positioning. Reads with a score lower than 30 were discarded which means that there is one in a thousand chance that a reported position is wrong.

### Statistical analysis of the different biases in the contact frequencies

In the following, we analyzed separately each different experiment conducted in [13] since different protocols can produce different results. Notably, the use of the secondary enzyme (MspI or MseI) change the potential interactions that can be observed.

Only the reads exhibiting a position on the genome reconcilable with the protocol design were retained (Figure 1A). Firstly, they are expected to map at a distance of about 20 bp to the nearest Hind III restriction site due to the use of the enzyme Ecop15I at the step 10 of the protocol (Figure 1A). We computed the number of read pairs as a function of the distance between the beginning of the read to the next RE1 site for each experiment. We found little difference between condition A and condition B (conditions A and B differ in the DNA concentration at the 3C step: A: 0.5 *μg*/*ml* , B: 0.3 *μg*/*ml* ). Whereas reads from datasets HindIII-MspI-A and HindIII-MseI-A have maximums for distances equals to 20, 21 and 22 bp, HindIII-MspI-B, HindIII-MseI-B and HindIII-MseI-uncross-control-B exhibit maximums for distances equals to 21, 22 and 23 bp (see Additional file 1: Figure S11). We only kept reads with distance between the beginning of the read and the next RE1 site equal to 20, 21 and 22 bp for condition A and equals to 21, 22 and 23 bp for condition B. Secondly, interactions involving fragments which have no restriction site for the secondary enzyme or a secondary site with a position located less than 20 bp from the first restriction site were also discarded. Finally, interactions corresponding to self-circularization (loops) and ligation of adjacent fragments (religation events) were removed from the analysis.

#### Bias of fragments sizes

The influence of the size of the RF on the observed frequency of interaction was analyzed as followed. Firstly, the sizes of each fragment were binned into equally sized windows (bin size: 100 bp). For each bin, the number of possible fragments *N*_*i*_ was counted according to the initial distribution of fragment sizes. The number of detected reads in the experiment *R*_*i *_is counted for each bin. Then, the number of reads per fragment *r*_*i*_ was calculated from these two numbers, with *r*_*i*_= *R*_*i*_/*N*_*i*_ . We fitted the data points with the following function: *f*(*x*)=*A*(1−(1−*p*_*c*_)^*x*^) which is related to the probability that the fragment is crosslinked at least one time. *A* is a normalization constant and *p*_*c*_ is the probability of crosslink by base paire (we found *A*≃4000 and *p*_*c*_≃0.004 ). The effect of the fragments size on the number of interaction reads before and after SCN is represented on Additional file 1: Figure S12 in the additional documentation.

#### Bias of GC content

The GC content influence was determined by binning the GC content of the mean of the two reads of each interaction (taking the sequence of the 20 bp before or after the restriction site RE1 according to the orientation of the read) into equally sized bins (bin size: 2.5%). For each bin, the number of possible interactions *N*_*i *_according to the initial distribution of GC contents, and the number of detected reads in the experiment *R*_*i*_ were estimated. These two numbers were divided to generate the number of reads per possible interaction: *r*_*i*_ = *R*_*i*_ / *N*_*i*_.

#### Bias in the circularization steps

The effect of the lengths of the DNA segment during circularization steps was analyzed by binning the size of the circularization segment into equally sized bins (bin size: 1 bp). The lengths were calculated using the coordinates of the positions of RE1 and RE2 restriction sites (MspI or MseI) on the reference genome. For each bin, the number of possible interactions *N*_*i *_according to the initial distribution of segment lengths and the number of detected reads in the experiment *R*_*i *_were estimated. These two numbers were divided to give the number of reads per possible interaction: *r*_*i*_ = *R*_*i*_/*N*_*i*_.

### Generation of matrices

Before the normalization step, we removed an important number of restriction fragments that could not be correctly detected in the experiment. First, non-mappable fragments were discarded. They correspond to fragments whose both extremities give ambiguous mapping (i.e the 20 bp sequence of the read can be located in several loci in the genome due to the presence of repeated sequences). 104 fragments felt into this category, most of them positioned in the subtelomeric regions of the chromosomes which are indeed enriched in repeated sequences. Second, all RFs that did not present a RE2 site were discarded (i.e. a MspI site for the experiment carried out with HindIII and MspI as RE1 and RE2, respectively). Intriguingly, these fragments are still detected in the experiment but with a smaller number of reads: Additional file 1: Figure S2 A represents the distribution of the number of reads per fragment. Two groups can be distinguished: a group corresponding to fragments that do not exhibit a secondary enzyme restriction site (having a number of reads inferior to 1000) and a second group corresponding to fragments having a RE2 site. Overall, 1217 RFs were concerned, which left 3098 RFs from the original 4454 for the MspI-HindIII experiment.

In addition, several RFs still exhibited a very small number of interaction reads with respect to the average (less than a few dozens reads re. the HindIII-MspI experiment), as seen on Additional file 1: Figure S2 B were the distribution of the euclidian norms of all fragments is plotted. Fragments with a norm under 30 were discarded from the analysis. 168 fragments felt into this category when considering inter-chromosomal interactions (see Additional file 1: Figure S2 B) and, in good agreement with the biases identified above, they exhibited either low GC content at their extremities, or the length of the two ligated fragments dA + dB had disfavored circularization.

Then each column vector was normalized to one, using the euclidian norm.

Then each line vector of the resulting matrix was normalized to one. The whole process was repeated sequentially until the matrix become symmetric again with each row and each column normalized to one. Convergence is not mathematically guaranteed for any matrix. For positive matrices which we have to deal with, it is generally attained in two or three iterations. For graphic representation the matrix was blurred using a convolution matrix, with as kernel the 3x3 matrix [0.05 0.05 0.05; 0.05 0.05 0.05; 0.05 0.05 0.05]. The convolution was repeated 10 times so that the structures appear clearly.

For the intra-chromosomal interactions, an extra step was added before normalization to take into account the effect of the genomic distance. First, we average the number of reads per possible interaction for every possible genomic distance. For each bin, the number of possible interactions *N*_*i*_ according to the initial distribution of genomic distances was estimated as well as the number of detected reads in the experiment *R*_*i*_. Then, these two numbers were divided to generate the number of reads per possible interaction: *r*_*i*_ = *R*_*i*_ / *N*_*i*_. Then, we use polynomial functions to fit the data points (see Additional file 1: Figure S13). Finally, we divide the number of reads of the experiment for each interaction by the expected value given by the fit at the genomic distance of the interaction.

This normalization step allows us to see interactions that are stronger than what it was expected due to the genomic distance effect. The SCN can be applied subsequently.

### Re-assessing the 3D colocalization of genomic elements

We used the statistical tool called Receiver Operating Curve (ROC) to look for 3D colocalization of several genomic elements. We slightly modified the initial method. We process as follows: first, we selected only the interactions containing one or two fragments containing the genomic element (centromere, telomeres, early origins of replication [25] or tRNA) instead of taking all detected interactions. We ranked the interactions of this set by p-values for the data of [13] and by the normalized interaction score for the normalized data. A interaction is labeled “positive” if both fragments contain the genomic element and negative in the other case. The ROC is generated by traversing the ranked list and plotting the percentage of positive and negative interaction above the threshold (p-value or normalized interaction score). If a genomic element tends to have strong interactions then the percentage of the positive interactions would be higher and the corresponding curve will be above the line x=y. Telomeres regions were determined as the last ten RF from each arm. Positions of early origins of replication and tRNA were similar to those used in [13].

## Competing interests

The authors declare no conflicts of interests.

## Author’s contributions

AC, RK and JM designed the analysis. AC and HMN performed the analysis. AC, HMN, MM, RK and JM interpreted the data. AC, RK and JM wrote the manuscript. All authors read and approved the final manuscript.

## Supplementary Material

Additional file 1 **Additional-documentation.** This document gives more information concerning the filtering of fragments and the normalization procedure. Click here for file
