# Supplementary material for: Normalization of a chromosomal contact map
Source: BMC Genomics. 2012 Aug 30;13:436. doi: 10.1186/1471-2164-13-436 (PMC3534615; doi:10.1186/1471-2164-13-436)
Supplement: Additional file 1 — Additional-documentation. This document gives more information concerning the filtering of fragments and the normalization procedure. [file 1471-2164-13-436-S1.pdf]

Additional documentation :

This file contains supplementary information concerning the filtering of fragments and the normalization procedure.

Figure S1 :

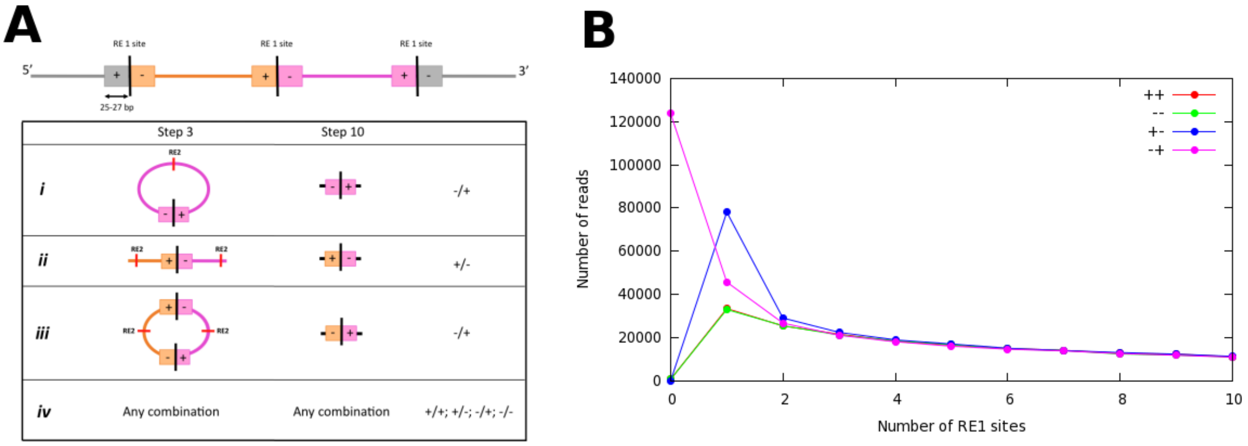

Schematic representation (A) and distribution (B) of the orientation of the reads obtained from different configurations of ligations between restriction fragments. A) Top: schematic representation of a chromosome, with restriction sites represented with black lines (RE1) and delimiting four RF (grey, orange, pink and grey). Bottom: the four different types of events expected as described in Figure 1A, step 3, are represented using the color code of the RF above. Step 3 and 10 are represented, with the positions of the second restriction enzymes identified with red lines. The expected orientation of the pair-end sequence reads are indicated in accordance with the schematic representation of the chromosome above. B) Distribution of the different pair-end sequence reads orientations recovered in the final bank, according to the number of RE1 sites separating the two ligated fragments.

Fgiure S2 :

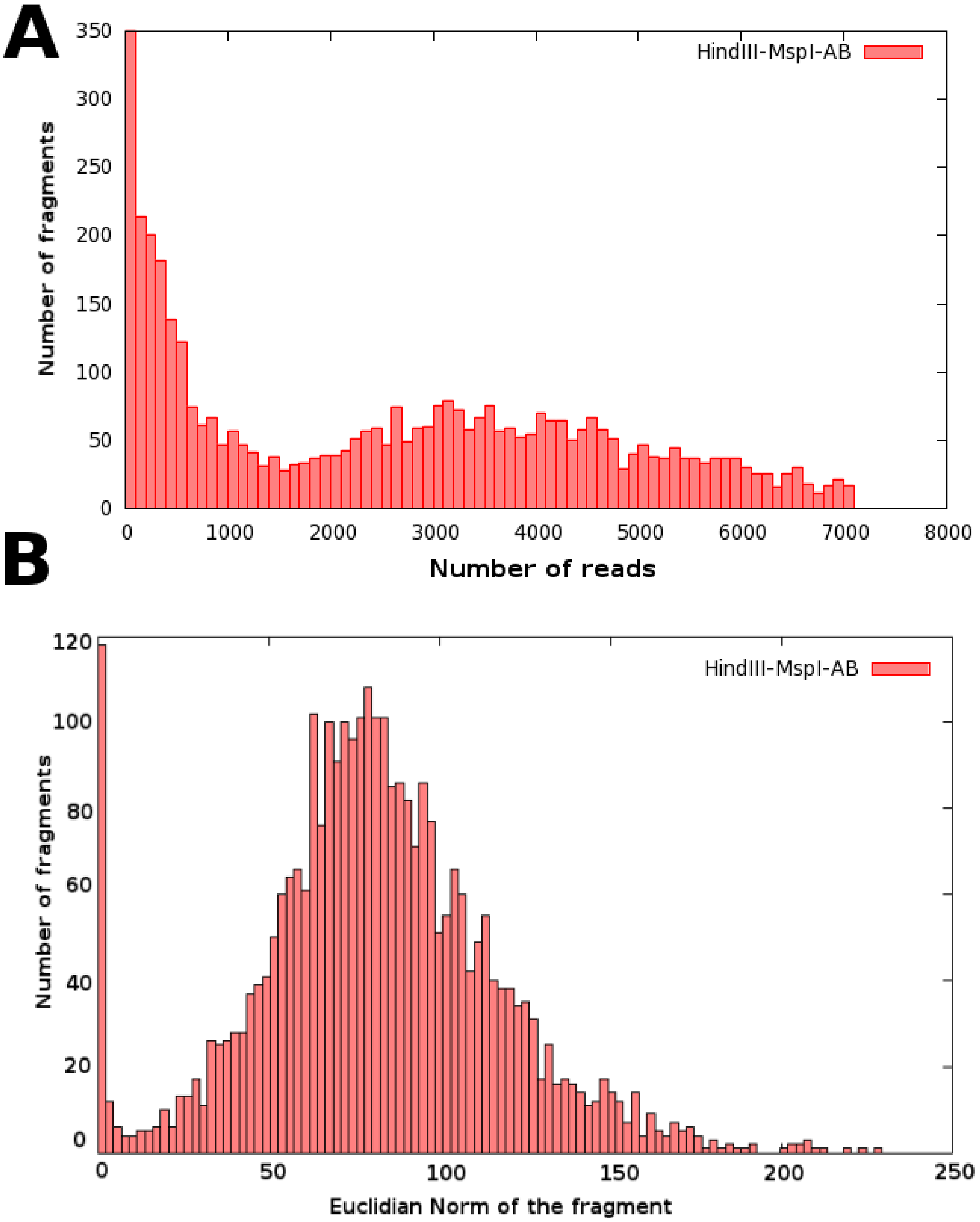

A) Distribution of reads per fragment for the inter chromosomal interactions in the experiment HindIII-MspI-Conditions AB showing two diffent groups. The first group corresponds to fragments that do not have a RE2 site.

B) Distribution of the norms for each fragment. It remains low interacting fragments that are removed.

Figure S3 :

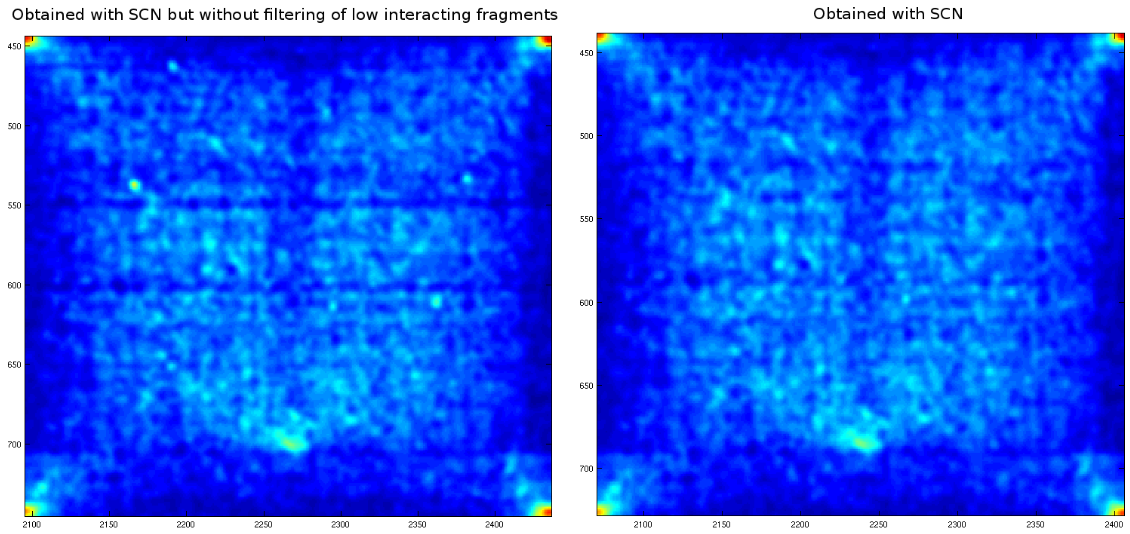

Comparison of matrices obtained with SCN procedure without (left) and with (right) filtering of low interacting fragments. Theses fragments were removed by plotting the distribution of the norms (see Figure S2B).
The picture shows a zoom of the matrices for interactions of chromosomes IV, XIII and XIV. The step of filtering low interacting fragments is necessary to have a less noisy matrix. Indeed, interspersed discrete dots appear in the matrix obtained through unfiltered data that do not correspond to any relevant biological feature, and that correspond to artifacts. These dots result only from the amplification through normalization of very few reads on a fragment.

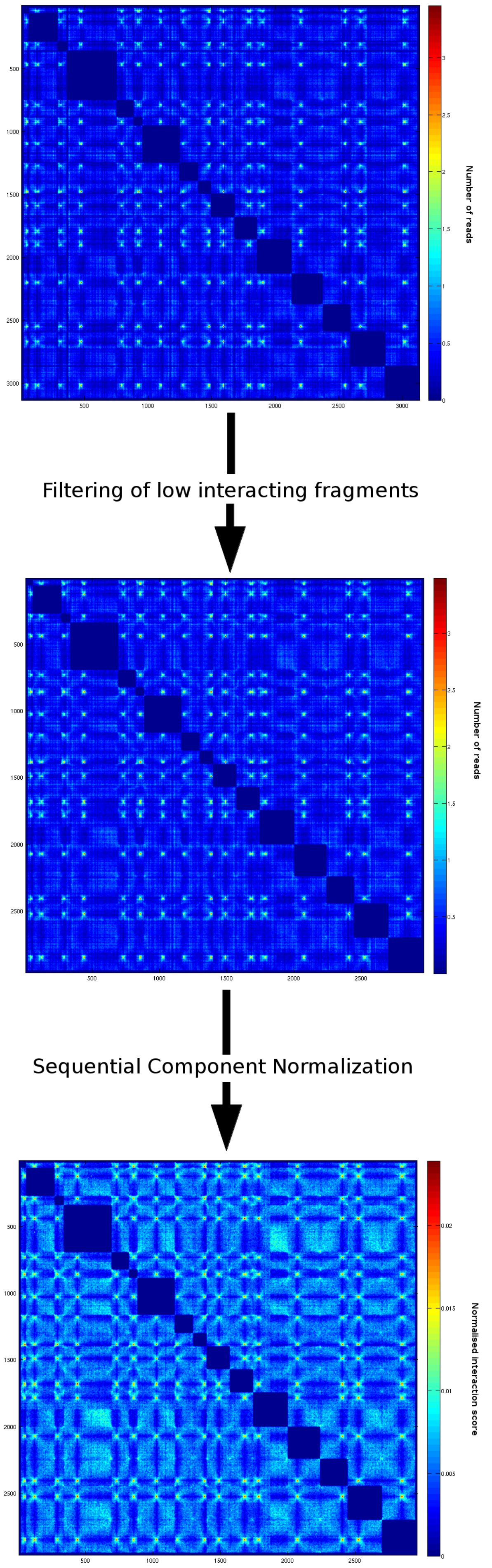

<u>Figure S4 :</u>
Inter-chromosomal contact maps for *S. cerevisiae* at diffent stages of the normalization procedure. The fisrt contact map represents interchromosomal interactions from raw data. In the second matrice, low interacting fragments have been removed. Finally, the last contact maps is the matrice obtained after processing with the SCN.

<u>Figure S5 :</u>

Matrices of interactions obtained with different normalization procedures.
We can see that the standard normalization produces a nosier matrix than the one obtained with SCN.

Matrix obtained with the standard normalisation $c^*_{ij} = \frac{c_{ij}}{\sum_k c_{ik} \sum_k c_{kj}}$

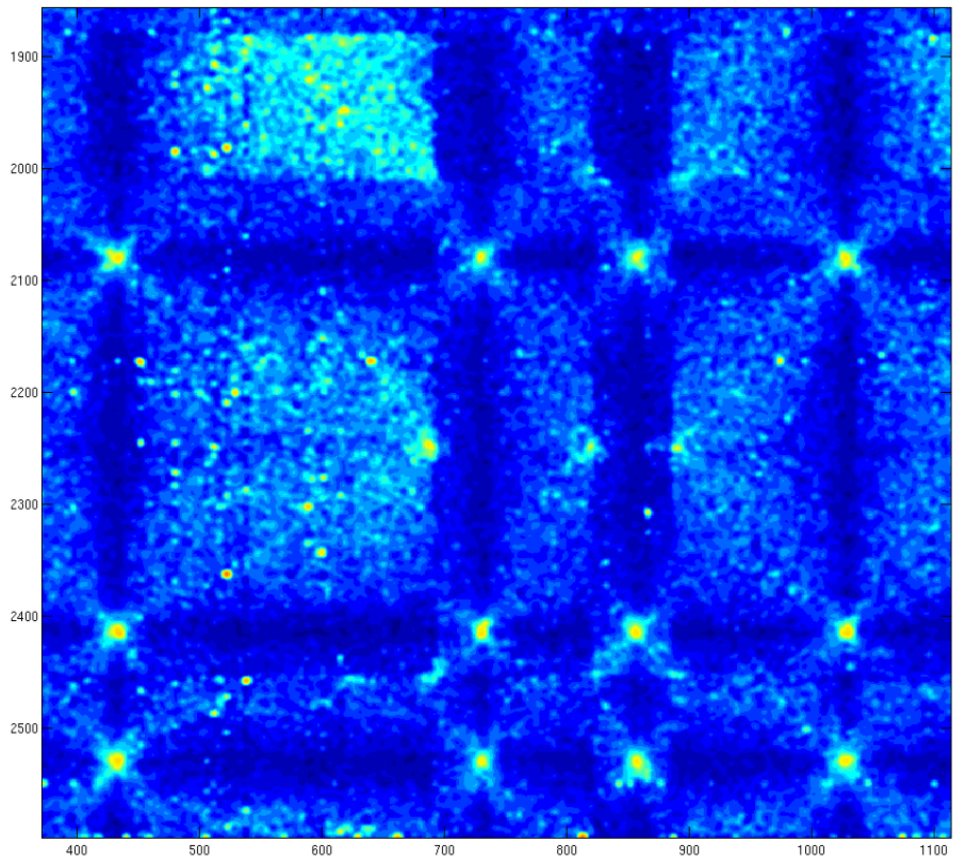

Matrix obtained with the SCN normalization

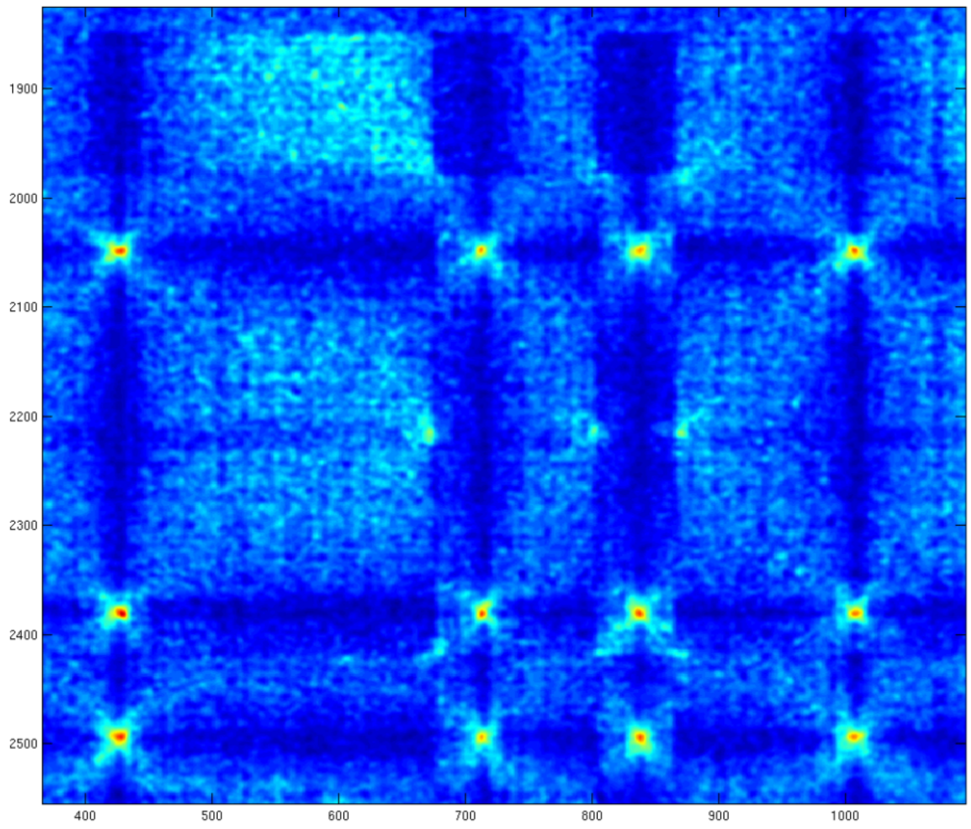

<u>Figure S6 :</u>

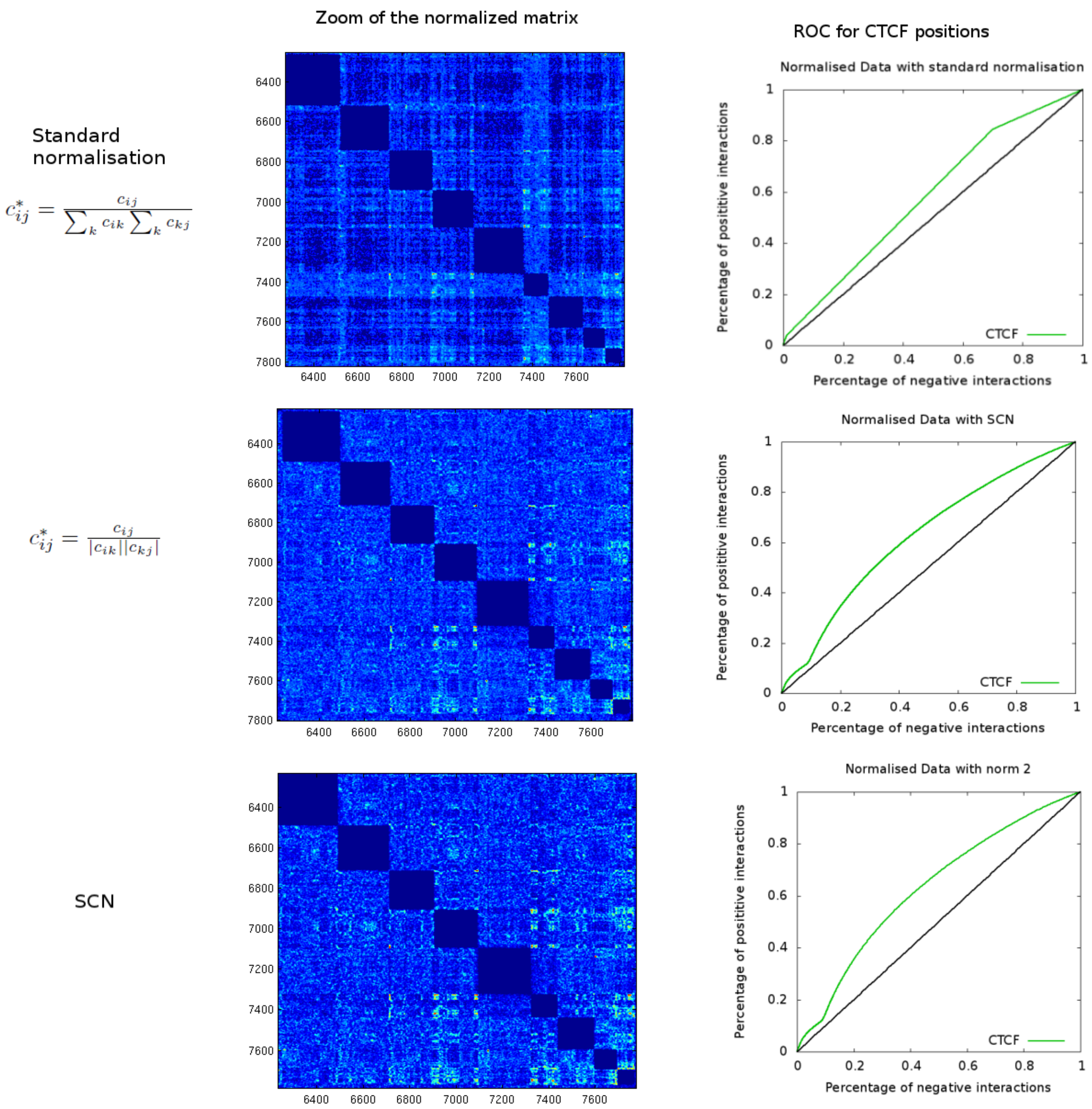

Comparison between the different norms used for the human contacts map.
A zoom of the matrices is shown for each type of normalization used.
The ROC test is applied on the normalized data for the positions of CTCF.
We can see that the matrix obtained with the standard normalization is less clear and the ROC gives a signal less pronounced than with the other types of normalization.

Figure S7 :

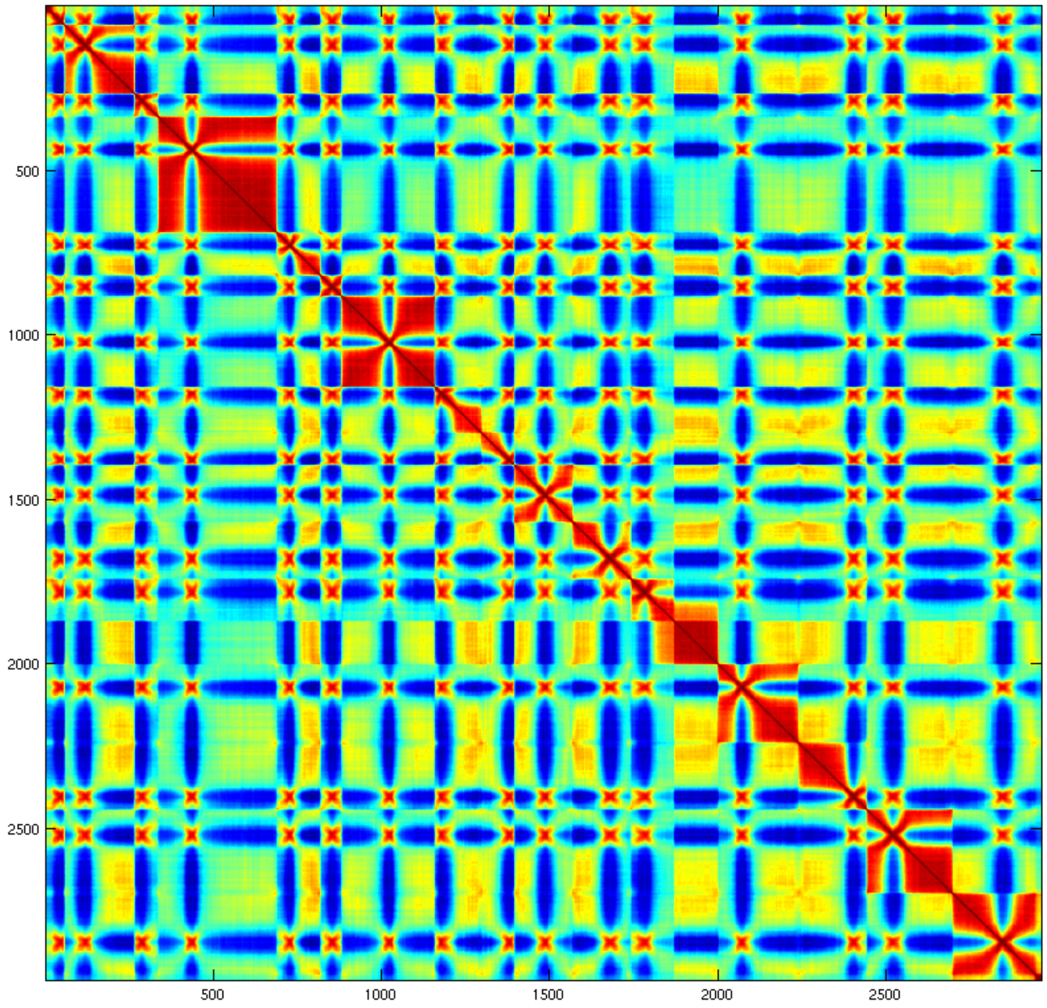

Correlation matrix for the yeast *S. cerevisiae* obtained with the data normalized with SCN for inter-chromosomal interactions . Each element of this matrix is the Pearson coefficient between the vectors i and j of the matrix of interactions. The same approach was used in [2].

Figure S8 :

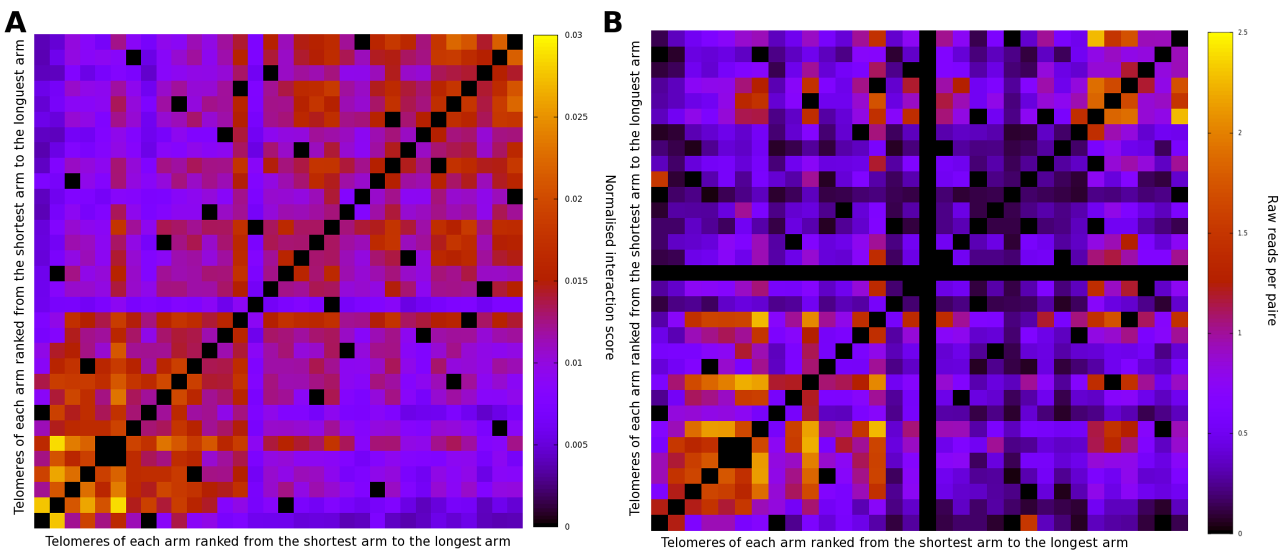

Representation of the normalized interaction score for each pair of subtelomeric regions (defined as the last 20 restriction fragments of each chromosome arm, and according to the reference genome).
Only detectable fragments were taken into account. Telomeres are ordonned as a function of the size of the chromosome arm they belong to.
A) normalized data. B) raw data (experiment HindIII-MspI-Conditions AB).

Figure S9 :

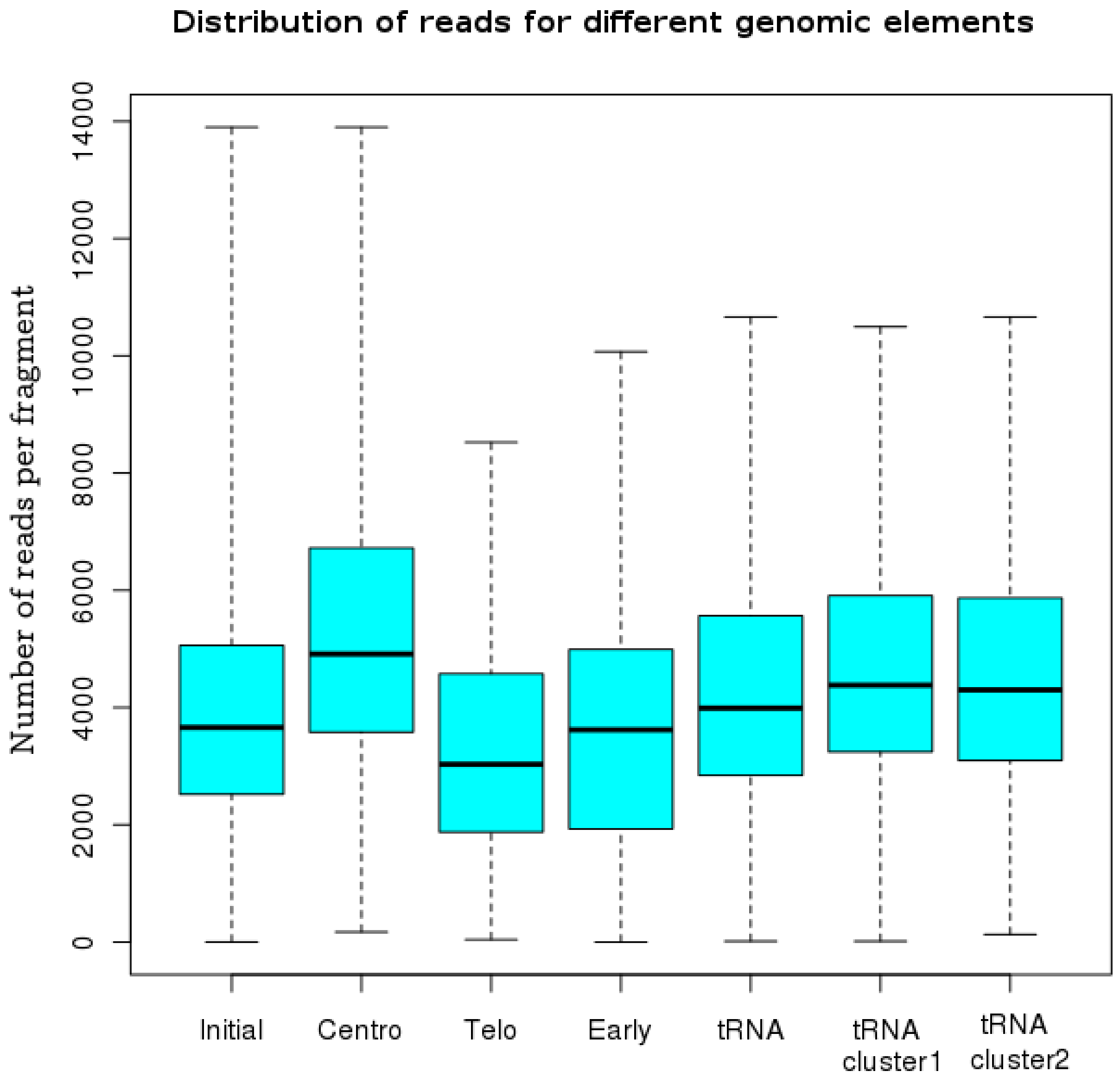

Box plots of the number of reads per fragment for the inter chromosomal interactions for different groups of fragments for the raw data (of the HindIII-MspI-AB experiment). The thick line gives the median of the distribution, the blue zone deliminates the first and third quartile and the whiskers give the extreme values of the distribution. 'Initial' refers to all detectable fragments, 'Centro' refers to the 10 fragments around centromers, 'Telo' refers to the 10 last fragments of each arm, 'Early' refers to the fragments containing at least one early origin of replication, 'tRNA' refers to the fragments containing at least one tRNA gene. 'tRNA cluster 1' refers to the fragments of the cluster 1 of tRNA proposed in [1]. 'tRNA cluster 2' refers to the fragments of the cluster 2 of tRNA proposed in [1].

Figure S10 :

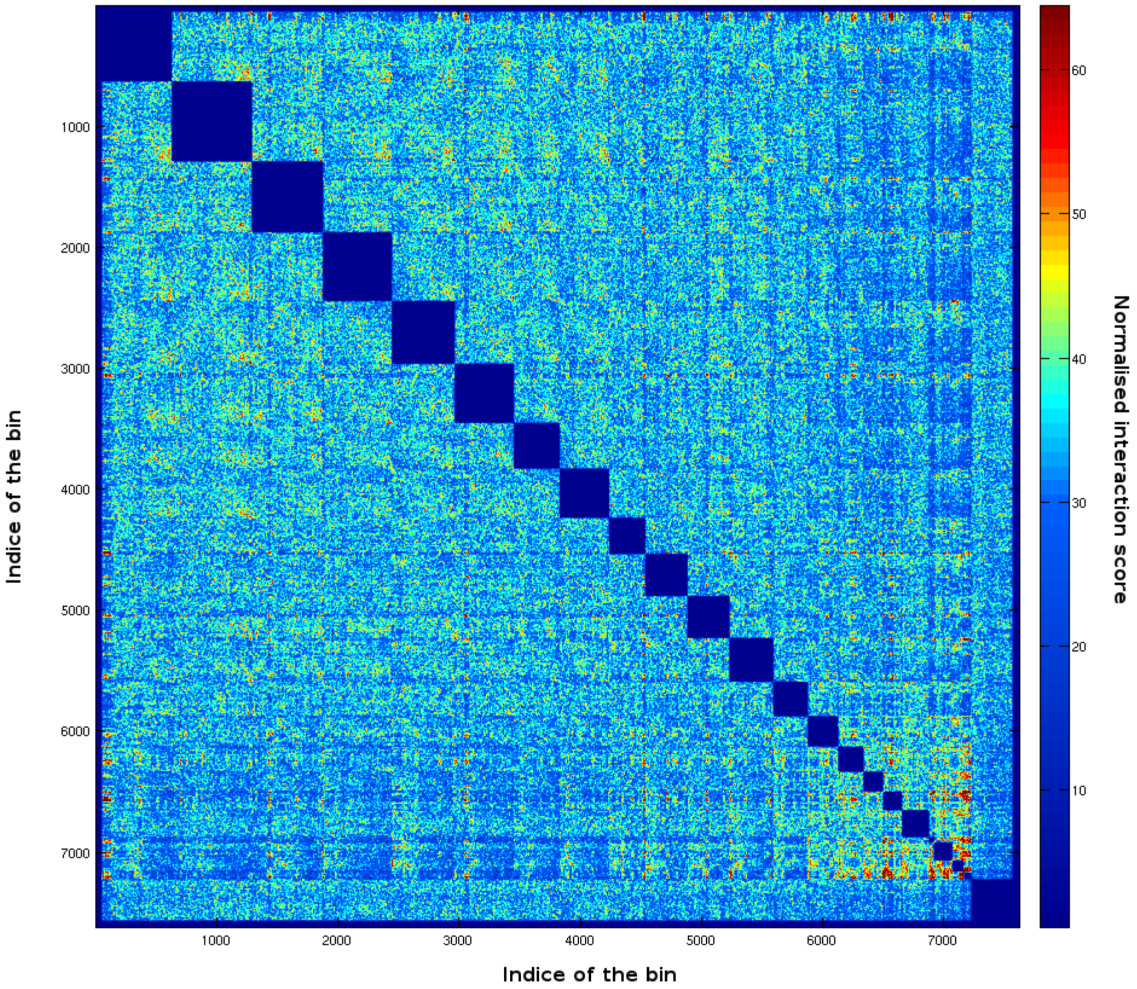

Inter-chromosomal contact map for the human genome obtained with the data of Lieberman et al. [2] and traited with SCN.
The color scale represents the normalized interaction frequencies between bins. Each bin contains 100 fragments. Chromosomes are ranked in the common order.

Figure S11 :

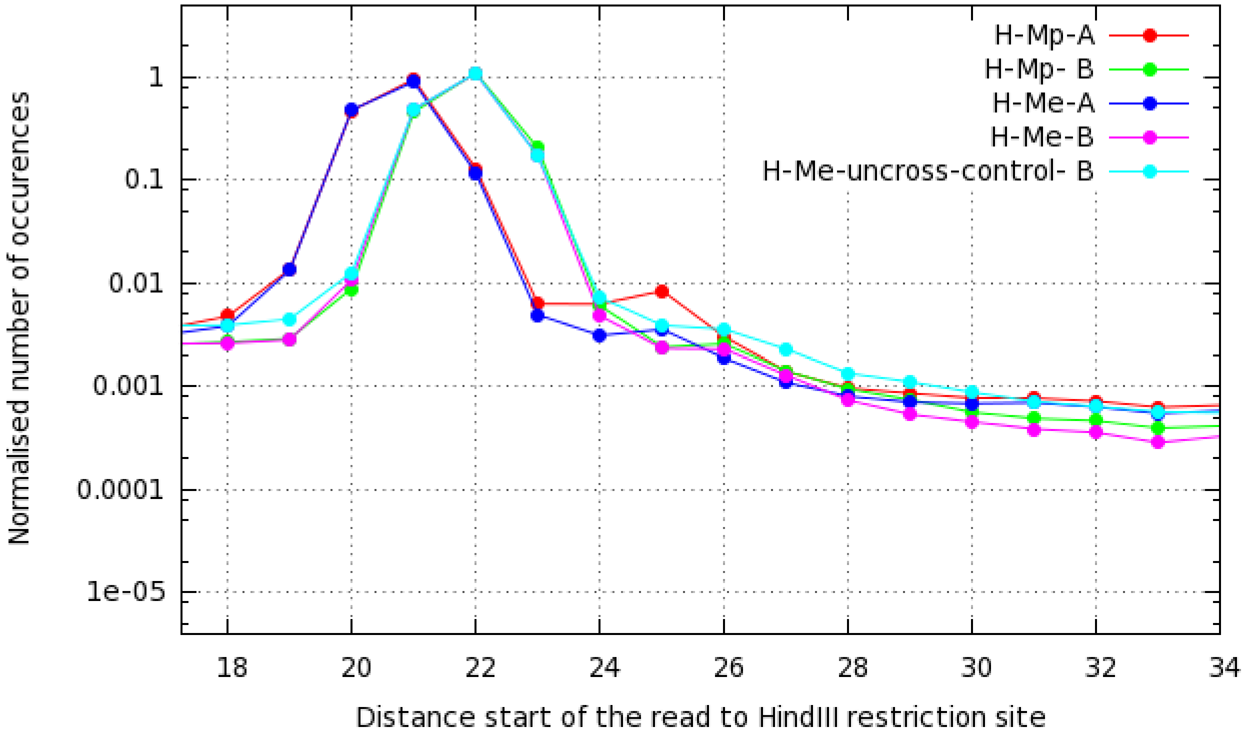

Normailzed number of occurences of the reads correctly aligned in fucntion of the distance between the beginning of the read and the next RE1 site.

Data are shown for each experiment and different conditions. Y axis is in log scale, most of reads are found at a distance of 20, 21, 22 bp for condition A and 21, 22, 23 bp for condition B.

Figure S12 :

A

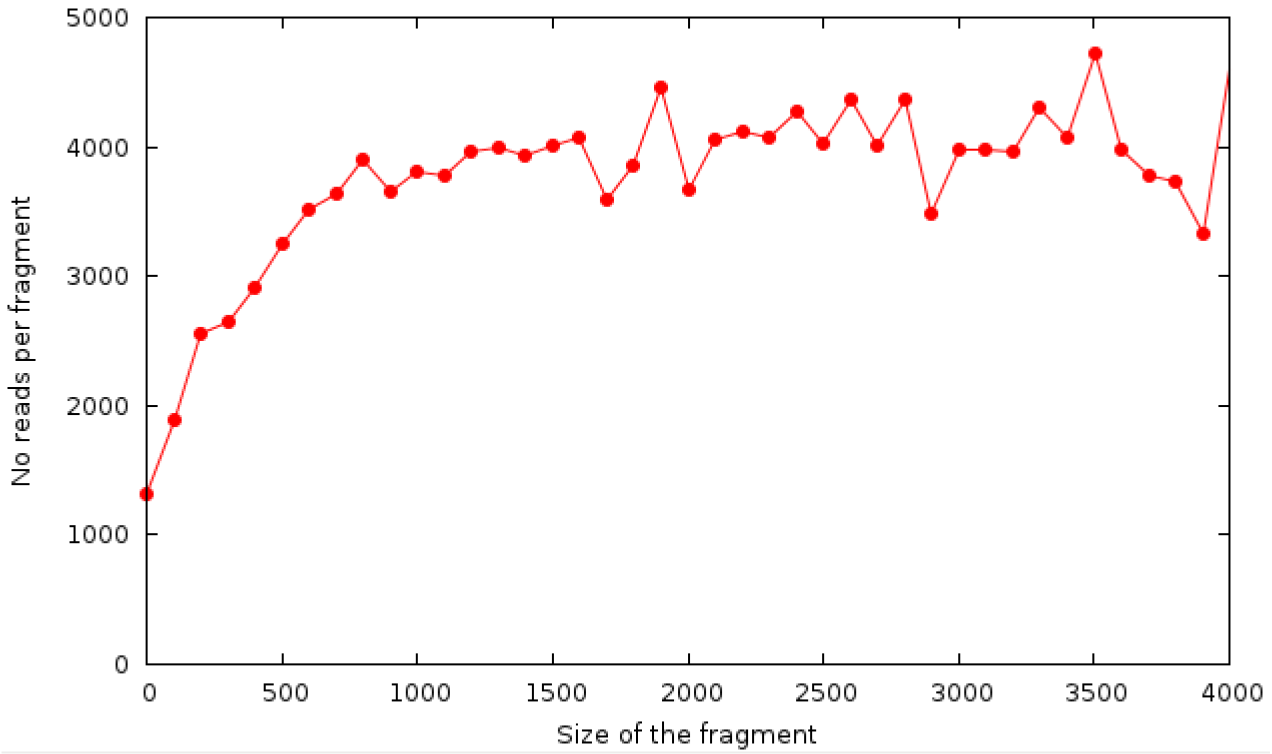

B

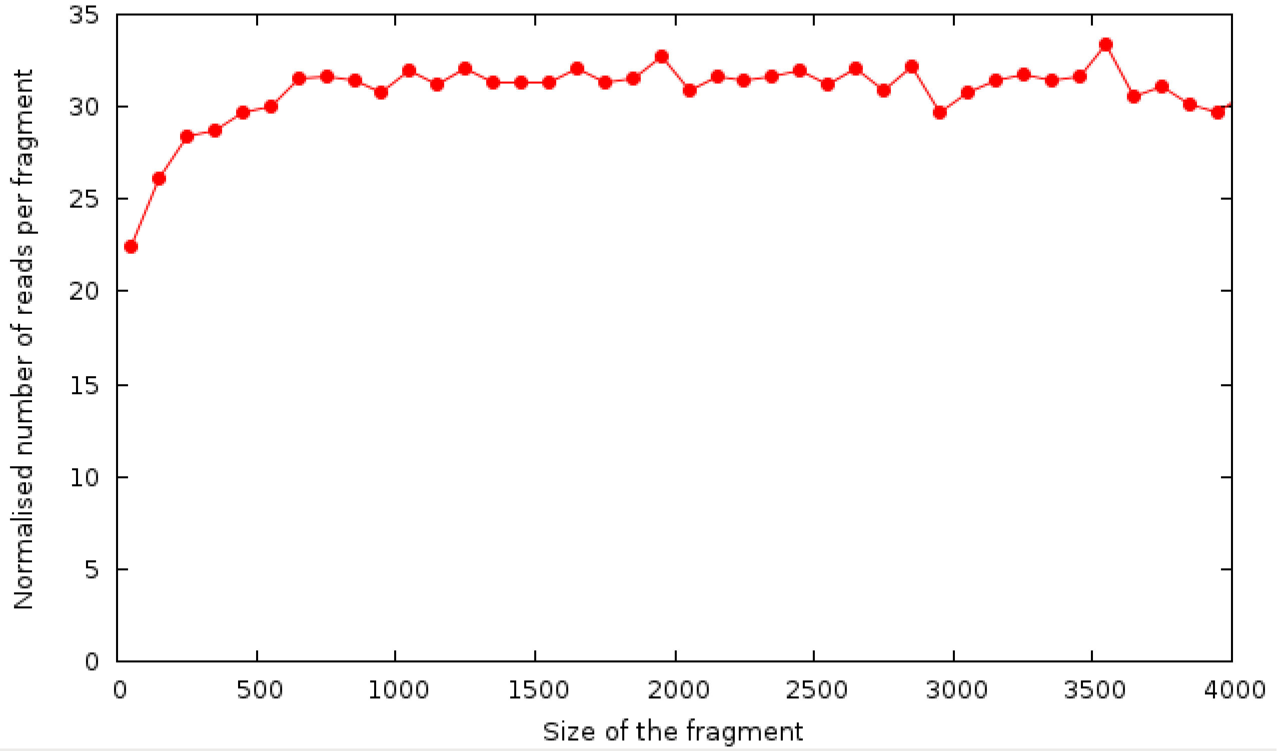

Effect of the fragment size on the number of interaction reads before (A) and after SCN (B). The representation is similar to the figure 1C of the main text.

Figure S13 :

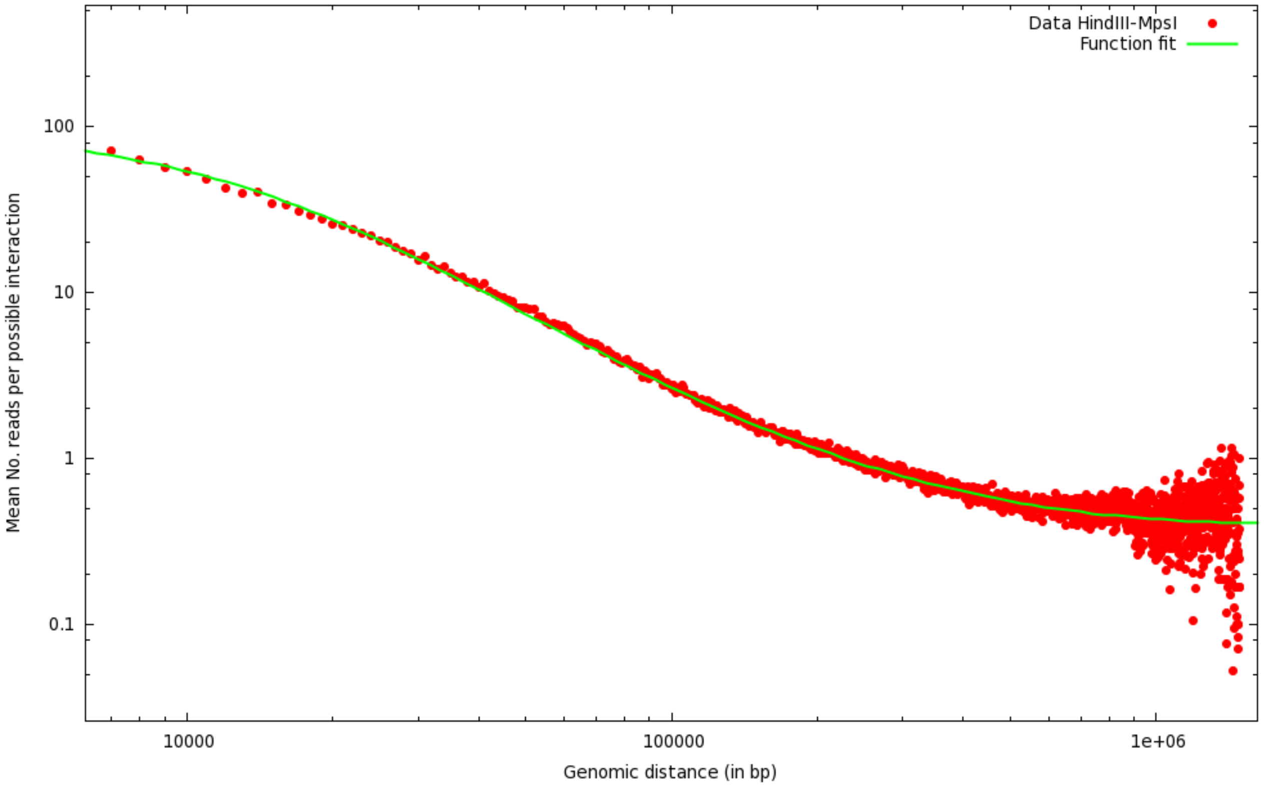

Number of reads per possible interaction as a function of the genomic distance separating the two extremities of the fragments from the experiment done with HindIII-MspI-Conditions AB (similar curve is obtained for the experiment performed with HindIII-MseI-Conditions AB). The data were fit with a polynomial function that was subsequently used to remove distance genomic effect for all intra-chromosomal interactions and to generate the matrices.

References :

[1] Zhijun Duan, Mirela Andronescu, Kevin Schutz, Sean McIlwain, Yoo Jung Kim, Choli Lee, Jay Shendure, Stanley Fields, C. Anthony Blau, and William S Noble. A three-dimensional model of the yeast genome. *Nature*, 465(7296):363–367, May 2010.

[2] Erez Lieberman-Aiden, Nynke L van Berkum, Louise Williams, Maxim Imakaev, Tobias Ragoczy, Agnes Telling, Ido Amit, Bryan R Lajoie, Peter J Sabo, Michael O Dorschner, Richard Sandstrom, Bradley Bernstein, M. A. Bender, Mark Groudine, Andreas Gnirke, John Stamatoyannopoulos, Leonid A Mirny, Eric S Lander, and Job Dekker. Comprehensive mapping of long-range interactions reveals folding principles of the human genome. *Science*, 326(5950):289–293, Oct 2009.
